# One-Step Combustion Synthesis of Carbon-Doped BiVO_4_ Yellow Pigments with Enhanced Visible-Light Photocatalytic Antibacterial Performance

**DOI:** 10.3390/molecules31122141

**Published:** 2026-06-17

**Authors:** Xiaojun Zhang, Tianxu Wang, Feng Jiang, Xiaoli Su, Xun Liu, Yanqiao Xu, Guo Feng, Qian Wu

**Affiliations:** 1 National Engineering Research Center for Domestic & Building Ceramics, Jingdezhen Ceramic University, Jingdezhen 333000, China; xuyanqiao@jci.edu.cn (Y.X.); fengguo@jci.edu.cn (G.F.); wuqian@jci.edu.cn (Q.W.); 2 State Key Laboratory of Advanced Environmental Technology & Guangdong Provincial Key Laboratory of Mineral Physics and Materials, Guangzhou Institute of Geochemistry, Chinese Academy of Sciences, Guangzhou 510640, China; liuxun@gig.ac.cn; 3 Department of Material Science and Engineering, Jingdezhen Ceramic University, Jingdezhen 333000, China; 13673104706@163.com

**Keywords:** pigment, yellow, bismuth vanadate, carbon doping, self-propagating combustion method

## Abstract

To integrate high chromaticity with visible-light-driven antibacterial functionality in yellow inorganic pigments, carbon-doped BiVO_4_ (C-BiVO_4_) pigments were synthesized via a one-step self-propagating combustion synthesis (SCS) using citric acid as a fuel and carbon source. The effects of citric acid dosage on phase composition, morphology, chromatic performance, and antibacterial activity were systematically investigated. The results indicate that carbon doping induces lattice expansion and oxygen vacancy formation, modulates the electronic band structure, and significantly suppresses photogenerated electron-hole recombination. At an optimal citric acid to BiVO_4_ molar ratio of 1.2, the pigment exhibits excellent yellow chromaticity (b* = 79.71). Under visible-light irradiation, C-BiVO_4_ achieves a methylene blue photodegradation rate of 96.63% and an *E. coli* inactivation efficiency of 99.99%, substantially outperforming undoped BiVO_4_. Moreover, the C-BiVO_4_ yellow pigment shows good dispersibility and thermal stability in PMMA and glass matrices and passes acute skin irritation and dermal toxicity tests, confirming its low toxicity and non-irritating nature. This work provides a new strategy for developing environmentally friendly inorganic pigments that combine high chromaticity with photocatalytic antibacterial functionality.

## 1. Introduction

Inorganic pigments, typically consisting of colorants and necessary additives, are indispensable components in various industrial fields due to their exceptional coloristic properties and chemical stability. They play a critical role in the decoration, protection, and performance enhancement of various commercial products [1]. With the rapid evolution of material science, there is an escalating demand to expand the functionality of inorganic pigments, moving beyond mere aesthetics to include smart features such as near-infrared reflective heat insulation, thermochromism, and photocatalytic antibacterial activity [2,3,4]. In the post-pandemic era, the increasing demand for environmental protection of indoor and workplace hygiene has driven the development of multifunctional inorganic pigments with high color-rendering properties and strong antibacterial activity to become a current research hotspot.

Photocatalytic antibacterial pigments, which impart color while simultaneously performing microbial inactivation, have garnered substantial attention due to their non-toxicity, stability, and ability to prevent the development of bacterial resistance [5]. Unlike traditional silver-based antibacterial materials, these pigments offer a cleaner and more sustainable approach, as they can decompose bacterial remains and toxins into CO_2_ and H_2_O under light irradiation. Furthermore, integrating these pigments into diverse substrates allows for the effective immobilization of the active powders, overcoming the separation and recycling challenges typically associated with loose photocatalysts [6]. While TiO_2_ (P25) is currently the commercial standard for such applications, its wide bandgap (Eg ≈ 3.2 eV) restricts its photocatalytic activity to the ultraviolet (UV) region, severely limiting its practical utility in indoor environments [7].

Bismuth vanadate (BiVO_4_), particularly in its monoclinic scheelite phase (m-BiVO_4_), has emerged as an ideal candidate due to its brilliant yellow hue, excellent chemical stability, and favorable visible-light response [8]. Despite these advantages, the practical application of m-BiVO_4_ is hindered by its relatively small surface area and the rapid recombination of photogenerated charge carriers, which limit its photocatalytic efficiency [9,10,11,12]. Extensive efforts have been devoted to improving its performance, including morphology control [13], heterojunction construction [14], noble metal deposition [15], and elemental doping [16,17,18]. However, these strategies often involve complex multi-step processes, costly precursors, or the introduction of toxic heavy metals, which may compromise the coloring performance or economic viability of the pigments. In this study, we propose a rational and facile one-step self-propagating combustion synthesis (SCS) strategy to engineer carbon-doped BiVO_4_ (C-BiVO_4_) pigments, utilizing citric acid (CA) as a multifunctional agent serving as both fuel and carbon source. Through this approach, high-performance yellow pigments were synthesized, achieving an impressive yellowness value (b* = 79) and a 99.99% bacterial inactivation rate against *E. coli* under visible-light irradiation. The influence of citric acid dosage on the phase composition, morphology, coloristic properties, and antibacterial efficacy was systematically investigated. This work not only provides a robust pathway for the sustainable synthesis of multifunctional inorganic pigments but also validates their exceptional application stability in diverse matrices, such as polymethyl methacrylate (PMMA) plastic and glass.

## 2. Results and Discussion

**Figure 1:**
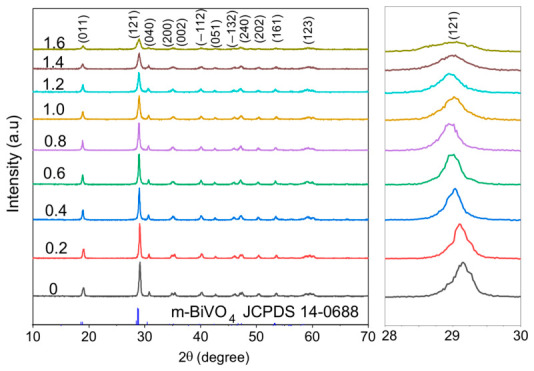
XRD patterns of C-BiVO_4_ pigments synthesized with varying citric acid amounts and the corresponding enlarged view of the (121) diffraction peak within the 2θ range of 28–30°.

To investigate the structural evolution and carbon-doping efficacy of BiVO_4_ synthesized via the self-propagating combustion method, this study employed XRD characterization on samples prepared with citric acid amounts ranging from 0 to 1.6 (Figure 1). The results confirmed the successful synthesis of the pure monoclinic phase BiVO_4_ (JCPDS 14-0688) across all samples while identifying a systematic shift of the (121) diffraction peak toward lower angles relative to the baseline of ~29.3° as the CA amount increased [19,20]. Furthermore, samples with higher CA amounts (1.2–1.6) exhibited slight peak broadening and intensity attenuation. These findings indicate that carbon incorporation induces lattice expansion and distortion via substitution or interstitial doping [21], thereby verifying the effective and controllable modulation of carbon doping in m-BiVO_4_ while suggesting that excessive CA dosage may result in reduced grain size and slight decreases in crystallinity. Additionally, the effects of heating rate and combustion atmosphere on the phase composition and chromatic performance of the C-BiVO_4_ pigments were further investigated, as shown in Figure S1 and Table S1. The results indicate that all samples synthesized in an air atmosphere at heating rates of 2–10 °C min^−1^ maintain the monoclinic BiVO_4_ phase with good crystallinity and high yellowness values (b* > 72). In contrast, the sample prepared under N_2_ atmosphere exhibits significant darkening in color and presents much lower chromaticity due to excessive carbon retention during combustion. These results confirm that the combustion atmosphere plays a critical role in regulating carbon content, crystallization behavior, and pigment color performance.

**Figure 2:**
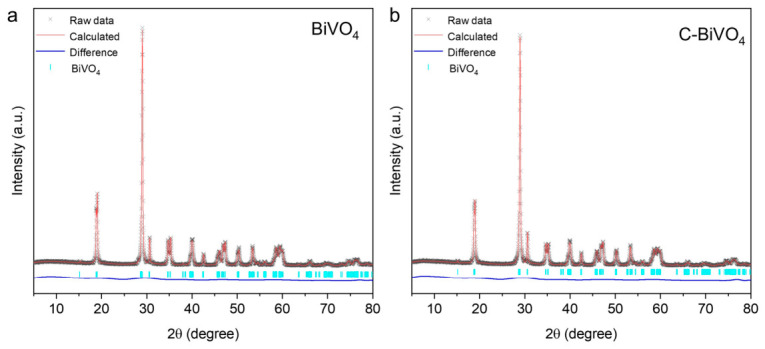
Refined XRD pattern corresponding to (**a**) the BiVO_4_ (n(CA)/n(Bi) = 0) and (**b**) C-BiVO_4_ (n(CA)/n(Bi) = 1.2) samples.

To further verify the incorporation of carbon into the BiVO_4_ lattice rather than the existence of surface residual carbon, Rietveld refinement was performed on the XRD patterns of pristine BiVO_4_ and C-BiVO_4_ (n(CA)/n(Bi) = 1.2), as shown in Figure 2 and Table 1. The refinement results exhibit good fitting reliability with low R_wp_ and R_p_ values, confirming the high crystallinity and phase purity of both samples. Compared with pristine BiVO_4_, the C-BiVO_4_ sample shows slight increases in lattice parameters from a = 5.1814 Å, b = 5.1033 Å, and c = 11.7019 Å to a = 5.1824 Å, b = 5.1113 Å, and c = 11.7042 Å, respectively. Consequently, the unit cell volume expands from 309.418 Å^3^ to 310.027 Å^3^. This lattice expansion is consistent with the shift of the (121) diffraction peak toward lower angles, which provides quantitative evidence for the incorporation of carbon atoms into the BiVO_4_ lattice, inducing lattice distortion rather than simply forming surface carbon residues.

**Figure 3:**
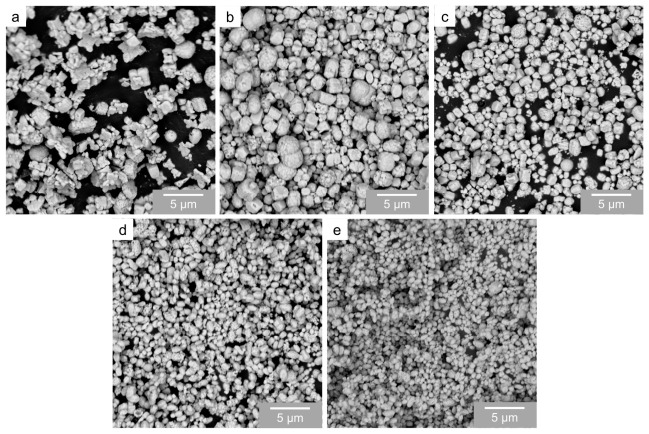
SEM images of C-BiVO_4_ pigments with different n(CA)/n(Bi) ratios: (**a**) 0, (**b**) 0.4, (**c**) 0.8, (**d**) 1.2, and (**e**) 1.6.

The morphologies of the C-BiVO_4_ pigments prepared with citric acid amounts ranging from 0 to 1.6 were investigated by SEM, as shown in Figure 3. The results reveal a systematic morphological evolution as the citric acid amount increases, transitioning from irregular sheets without citric acid to well-defined cubes (n(citric acid)/n(BiVO_4_) = 0.4–0.8) and finally to ellipsoidal structures (n(citric acid)/n(BiVO_4_) = 1.2–1.6), accompanied by a noticeable decrease in particle size and enhanced dispersion. This morphological transformation is attributed to the dual role of citric acid: as a fuel, it governs combustion dynamics and nucleation density [22]. As a carbon source, it alters crystal growth kinetics through doping [23]. Consequently, these findings demonstrate that the self-propagating combustion method enables precise morphological control over C-BiVO_4_ by adjusting the citric acid amount.

**Figure 4:**
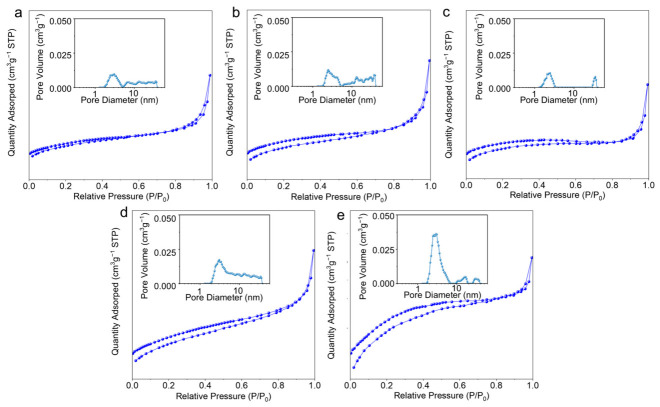
N_2_ adsorption–desorption isotherms and corresponding pore size distribution curves (insets) of C-BiVO_4_ pigments with different n(CA)/n(Bi) ratios: (**a**) 0, (**b**) 0.4, (**c**) 0.8, (**d**) 1.2, and (**e**) 1.6.

Figure 4 shows the N_2_ adsorption–desorption isotherms and corresponding pore size distribution curves of the C-BiVO_4_ pigments prepared with different n(CA)/n(Bi) ratios. All samples exhibit type-IV isotherms with H3-type hysteresis loops, indicating the presence of mesoporous structures formed by the aggregation of primary particles. With increasing citric acid amount, the BET surface area gradually increases from 4.6926 m^2^ g^−1^ for pristine BiVO_4_ to 4.7634, 5.6475, 8.6198, and 16.7481 m^2^ g^−1^ for C-BiVO_4_ samples with n(CA)/n(Bi) ratios of 0.4, 0.8, 1.2, and 1.6, respectively. This trend is highly consistent with the SEM observations, where the particle morphology evolves from irregular sheet-like structures to olive-like ellipsoidal particles with smaller sizes.

**Figure 5:**
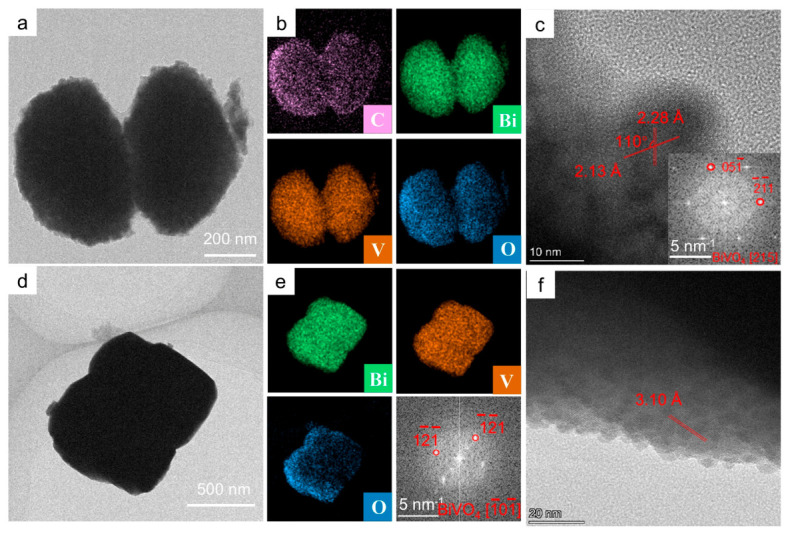
TEM images, EDS mappings, and HRTEM images of C-BiVO_4_ (**a**–**c**) and BiVO_4_ (**d**–**f**) pigments.

Figure 5 illustrates the TEM, EDS mapping and HRTEM images of C-BiVO_4_ and BiVO_4_ pigments. The results show that C-BiVO_4_ pigment exhibits a rough, ellipsoidal morphology, approximately 400–500 nm in size (Figure 5a), with EDS mapping confirming the uniform distribution of carbon within the matrix (Figure 5b). HRTEM combined with FFT analysis measures lattice spacings of 2.28 Å and 2.13 Å with an included angle of 110°, corresponding to the $\left(\bar{2}\bar{1}1\right)$ and $\left(05\bar{1}\right)$ planes and a zone axis of [215], confirming that the product is monoclinic m-BiVO_4_ (Figure 5c). In contrast, the pure BiVO_4_ sample (Figure 5d–f) exhibits an irregular sheet-like morphology with sharp edges and larger particle sizes (approximately 600–800 nm). No carbon signal is detected by EDS mapping, confirming the absence of carbon doping. HRTEM shows a lattice spacing of 3.10 Å corresponding to the $\left(\bar{1}\bar{2}1\right)$ plane with a zone axis of [$\bar{1}0\bar{1}$], also identified as monoclinic m-BiVO_4_ [24]. Additional HRTEM images (Figure S2) reveal clear lattice fringes and good crystallinity of the C-BiVO_4_ particles, while no obvious amorphous carbon shell or continuous surface carbon coating layer was observed around the particles. These findings demonstrate that citric acid acts as both a morphology-directing agent and a carbon source, inducing a transition from sheet-like to ellipsoidal morphology and facilitating uniform carbon doping [25].

**Figure 6:**
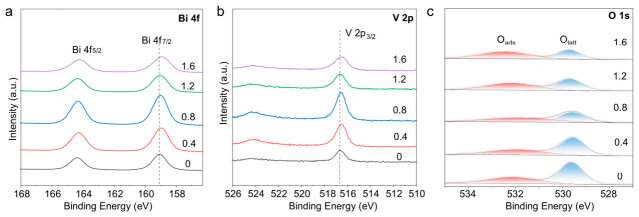
XPS core level spectra of C-BiVO_4_ pigment with varying citric acid amounts: (**a**) Bi 4f, (**b**) V 2p and (**c**) O 1s.

To elucidate the influence of carbon doping on the electronic structure of BiVO_4_ and determine the underlying doping mechanism, X-ray photoelectron spectroscopy (XPS) was employed to characterize samples prepared with varying citric acid amounts (Figure 6). The results demonstrate that, with increasing carbon content, both Bi 4f and V 2p peaks exhibit a systematic negative shift toward lower binding energies (relative to the baseline values of ~159.1 eV and ~516.7 eV for pure BiVO_4_), indicating a significant modulation of the local electronic environment surrounding Bi and V atoms [16,26,27]. Concurrently, the O 1s spectra reveal a relative decrease in the lattice oxygen signal (~529.5–530.0 eV) alongside a notable increase in the oxygen vacancy-related adsorbed oxygen signal (~531.5–532.5 eV) [28,29]. Consequently, it is concluded that carbon atoms have been successfully doped into the BiVO_4_ lattice, inducing lattice distortion and simultaneously generating oxygen vacancies to maintain charge neutrality.

**Figure 7:**
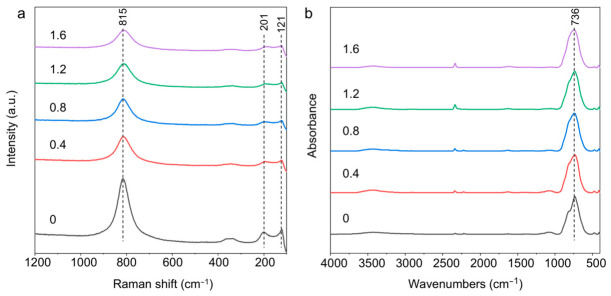
Raman (**a**) and FTIR (**b**) spectra of C-BiVO_4_ pigment with varying n(CA)/n(Bi) ratios.

Figure 7 presents the Raman and FTIR spectra of C-BiVO_4_ pigments prepared with different citric acid amounts to further elucidate the influence of carbon doping on the local structure and chemical bonding environment of BiVO_4_. In the Raman spectra (Figure 7a), all samples exhibit the characteristic vibrational modes of monoclinic scheelite BiVO_4_, confirming that the crystal structure remains unchanged after carbon doping. The strong Raman peak located at ~815 cm^−1^, is assigned to the symmetric stretching vibration of the V–O bond in the VO_4_ tetrahedron [30,31]. The peak gradually shifts toward lower wavenumbers with increasing citric acid amount, accompanied by slight peak broadening. This phenomenon indicates the weakening and distortion of V–O bonds caused by lattice expansion and local structural disorder induced by carbon incorporation [32,33].

The FTIR spectra shown in Figure 7b further support these structural changes. The characteristic absorption band located at ~736 cm^−1^ [34] corresponds to the asymmetric stretching vibration of V–O bonds in monoclinic BiVO_4_, which exhibits a slight blue shift with increasing carbon content, indicating the change in the local bonding environment due to carbon-induced lattice distortion and defect generation. Meanwhile, the gradual weakening of the shoulder near ~830 cm^−1^ is consistent with the Raman results, further confirming the structural perturbation of the VO_4_ units [34,35]. Furthermore, the Raman and FTIR results correlate well with the SEM, BET, and XPS analyses. The increased structural disorder induced by carbon doping suppresses crystal growth during combustion synthesis, leading to the morphology evolution from irregular sheet-like particles to uniform ellipsoidal structures with a smaller size, accompanied by an increase in BET specific surface area. Meanwhile, the lattice distortion revealed by Raman, FTIR and XPS contributes to the enhanced visible-light absorption and improved charge separation behavior. Consequently, the synergistic modulation of crystal structure, morphology, and electronic properties is expected to enhance the photocatalytic antibacterial performance of the C-BiVO_4_ pigments.

**Figure 8:**
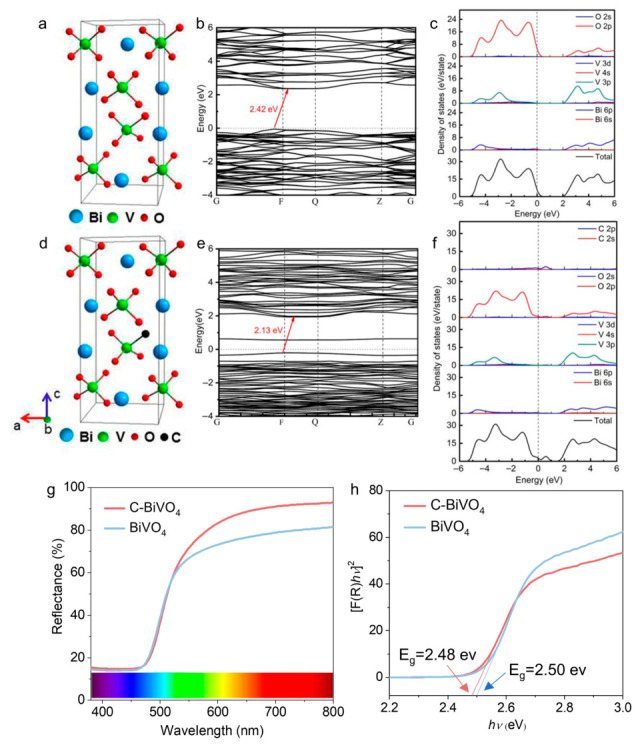
DFT calculations and experimental optical analysis. (**a**,**d**) Optimized crystal structures, (**b**,**e**) electronic band structures, (**c**,**f**) total and partial density of states (DOS and PDOS) and (**g**,**h**) UV-vis diffuse reflectance spectra and the corresponding plots of (F(R)*hν*)^2^ vs. *hν* of BiVO_4_ and C-doped BiV(O_0.95_C_0.05_)_4_, respectively. (In Figure 8(**d**), a, b, and c represent the crystallographic axes).

To investigate the effect of carbon doping on the crystal structure, electronic properties, and optical performance of BiVO_4_, density functional theory (DFT) calculations were performed and compared with experimental results. Firstly, crystal structure models of pristine BiVO_4_ and C-doped BiVO_4_ were constructed (C partially substituting the O site), and their band structures and density of states (DOS) were calculated, as shown in Figure 8a–f. Subsequently, corresponding samples were synthesized via a self-propagating combustion method and experimentally characterized using UV-vis diffuse reflectance spectra (Figure 8g,h). DFT calculations revealed that after C doping, the crystal retains a monoclinic scheelite structure with slight lattice distortion, the band gap decreases from 2.42 eV for pristine BiVO_4_ to 2.13 eV, and the DOS analysis shows that C 2p orbitals introduce impurity states within the band gap. In contrast, UV-Vis DRS experimentally determined the band gaps of pristine BiVO_4_ and C-BiVO_4_ to be 2.50 eV and 2.48 eV, respectively, with a band gap reduction of only 0.02 eV. The calculated bandgap of C-BiVO_4_ is 2.13 eV, which is lower than the experimentally measured value (2.48 eV). This discrepancy is mainly attributed to the intrinsic theoretical bandgap underestimation (TBE) associated with conventional GGA-based DFT calculations for semiconductor systems [21,24]. Nevertheless, both the DFT calculations and experimental UV-Vis results consistently demonstrate that carbon incorporation leads to the narrowing of the bandgap and thus enhances the visible-light absorption of BiVO_4_.

**Figure 9:**
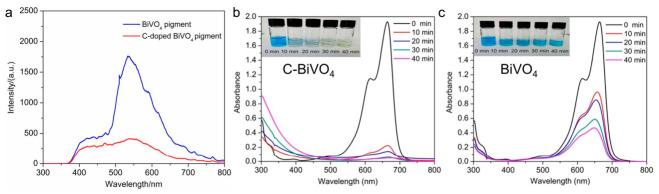
(**a**) PL spectra of BiVO_4_ and C-doped BiVO_4_ pigments. Time-dependent UV-vis absorption spectra of MB solutions during photocatalytic degradation using C-BiVO_4_ (**b**) and BiVO_4_ (**c**) pigments as photocatalysts (the insets display the corresponding visual color changes in the MB solutions over the degradation process).

Figure 9 presents comprehensive photoluminescence (PL) spectroscopy and photocatalytic degradation evaluations of methylene blue (MB) under simulated solar light. PL characterization revealed that the photoluminescence intensity of C-BiVO_4_ pigment (n(CA)/n(Bi) = 1.2) decreased significantly to approximately 20% of that of BiVO_4_ with the same emission peak position (~520 nm), indicating effective suppression of electron-hole recombination by carbon doping [36]. Correspondingly, photocatalytic performance tests demonstrated that after 40 min of irradiation, the MB degradation rate of C-BiVO_4_ reached 96.63%, markedly outperforming the 75.67% achieved by BiVO_4_ pigment.

**Figure 10:**
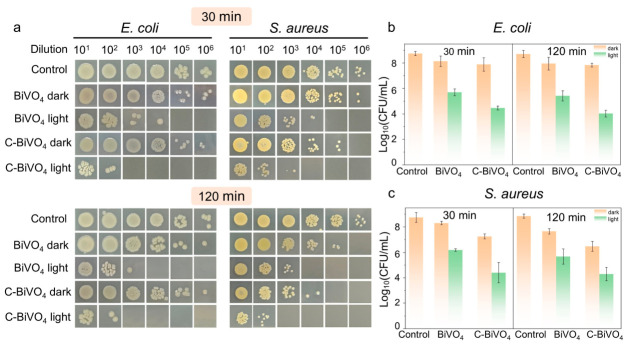
Antibacterial performance evaluation of the samples. (**a**) Digital photographs of bacterial colony formation on agar plates after treatment with BiVO_4_ or C-BiVO_4_ under dark and light conditions for 30 and 120 min, with corresponding bacterial viability expressed as Log_10_(CFU mL^−1^) for *E. coli* (**b**) and *S. aureus* (**c**). Error bars represent standard deviations from three independent experiments.

To evaluate the photocatalytic activity of the C-BiVO_4_ yellow pigment, antibacterial experiments were conducted against both Gram-negative *Escherichia coli* (*E. coli*) and Gram-positive *Staphylococcus aureus* (*S. aureus*) using a standard colony-forming unit (CFU) assay under dark and visible-light irradiation, as shown in Figure 10. The samples were incubated with bacterial suspensions for 30 and 120 min, followed by serial dilution and colony counting. Under dark conditions, both BiVO_4_ and C-BiVO_4_ exhibited limited antibacterial activity, indicating that the bacterial inactivation is mainly driven by photocatalytic processes rather than intrinsic toxicity. Upon visible-light irradiation, however, the bacterial viability decreased dramatically. For *E. coli*, the Log_10_(CFU mL^−1^) value of the C-BiVO_4_ group decreased from approximately 7.8 under dark conditions to ~4.5 after 30 min of irradiation and further to ~4.0 after 120 min, achieving a substantial bacterial reduction of over 99.9%. Similarly, the bacterial viability of *S. aureus* decreased from ~7.3 to ~4.7 after 30 min of irradiation and further to ~4.5 after 120 min. In comparison, pristine BiVO_4_ exhibited significantly weaker antibacterial efficacy under identical conditions. These results demonstrate that carbon doping markedly enhances the photocatalytic antibacterial performance of BiVO_4_ against both bacterial strains.

To ensure statistical reliability, all CFU experiments were performed in triplicate, and the corresponding results are presented as mean values with standard deviation error bars in Figure 10b,c. The relatively small error bars indicate good reproducibility and statistical reliability of the antibacterial experiments. Furthermore, the consistent antibacterial enhancement observed for both bacterial strains confirms that the photocatalytic antibacterial activity of C-BiVO_4_ is non-specific, thereby broadening the practical applicability of the pigment in antibacterial materials.

In addition, cyclic antibacterial experiments were performed to evaluate the long-term stability and reusability of the C-BiVO_4_ pigment under visible-light irradiation (Figure S3). The antibacterial efficiencies against *S. aureus* remained approximately 99.99%, 99.99%, 99.90%, 99.78%, and 99.47% after the 1st to 5th cycles, respectively, while the corresponding efficiencies against *E. coli* were maintained at about 99.99%, 99.99%, 99.94%, 99.90%, and 99.50%. Although a slight decrease in antibacterial activity was observed after repeated cycling, the antibacterial efficiencies toward both bacterial strains remained above 99.4% after five cycles. These results indicate that the C-BiVO_4_ pigment possesses excellent photocatalytic stability.

**Figure 11:**
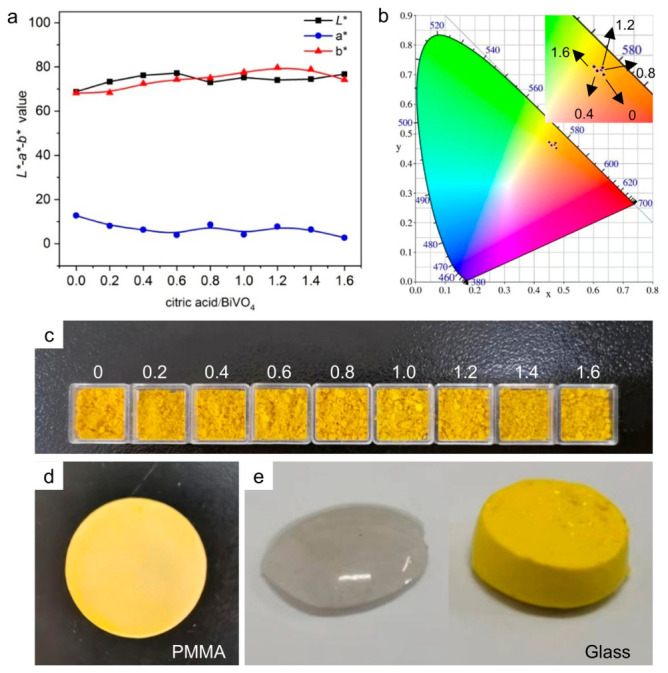
Optical properties and application of C-BiVO_4_ pigment. (**a**) CIE L*-a*-b* value with citric acid/BiVO_4_ molar ratio. (**b**) CIE chromaticity diagram. (**c**) Digital photograph of C-BiVO_4_ pigments. (**d**,**e**) Digital photographs of C-BiVO_4_ pigment applied on PMMA and glass (600 °C) substrates, respectively.

The influence of the citric acid amount on the chromatic performance and practical applicability of C-BiVO_4_ pigments is shown in Figure 11a–c. The results reveal that the yellowness (b*) of the pigments exhibits a trend of increasing first and then decreasing, among which the C-BiVO_4_ pigment (n(CA)/n(Bi) = 1.2) exhibits the deepest yellow with optimal coloristic attributes of L* = 74.09, a* = 7.78, and b* = 79.71 (Table S2). Conversely, excessive citric acid amount (>1.2) results in decreased color saturation due to carbon residues, which introduce gray-black impurities. Furthermore, application tests demonstrate that the pigments prepared under these optimized conditions exhibit excellent dispersibility, thermal stability, and coloring performance in both PMMA and glass matrices. Thus, C-BiVO_4_ yellow pigment successfully achieves the dual-function integration of “high color rendering and high antibacterial activity,” providing an effective strategy for developing inorganic pigments that combine aesthetic appeal with functionality.

Moreover, to evaluate the biological safety and practical applicability of the C-BiVO_4_ yellow pigment for consumer-related sectors, acute skin irritation and dermal toxicity tests were conducted in accordance with GB/T 21604-2008 and GB/T 21606-2008 standards (Table S3 and Table S4), respectively [37,38]. The experimental results demonstrated that in the skin irritation test, the scores for erythema and edema at all observation points were 0, well below the threshold of <0.5, classifying the pigment as non-irritating. Meanwhile, in the acute dermal toxicity test, neither toxic symptoms nor mortality was observed in SD rats within a 14-day period following a dosage of 2000 mg/kg, with an LD_50_ value greater than 2000 mg/kg, which categorized the substance as low toxicity. These findings confirm that the synthesized C-BiVO_4_ pigment, which exhibits both high chromaticity and excellent photocatalytic antibacterial performance, possesses superior biocompatibility and safety, providing a robust scientific basis for its potential application in consumer goods such as toys, surface coatings, and food packaging.

## 3. Materials and Methods

### 3.1. Sample Preparation

The bismuth and vanadium sources were bismuth nitrate pentahydrate (Bi(NO_3_)_3_·5H_2_O, 99%, Aladdin Co., Ltd., Shanghai, China) and ammonium metavanadate (NH_4_VO_3_, 99%, Aladdin Co., Ltd., Shanghai, China), respectively, while citric acid (C_6_H_8_O_7_·H_2_O, 99.5%, Aladdin Co., Ltd., Shanghai, China) was employed as both the fuel and carbon source. Deionized water was used as the solvent, while aqueous ammonia and nitric acid were employed for pH adjustment. Briefly, specific amounts of Bi(NO_3_)_3_·5H_2_O and citric acid were dissolved in 1 mM HNO_3_ solution. Simultaneously, NH_4_VO_3_ was dissolved in a separate 1 mM HNO_3_ solution. The vanadium precursor solution was slowly added dropwise into the bismuth precursor solution under continuous stirring to form a transparent green solution, with the molar ratios n(CA)/n(Bi^3+^)/n(V^5+^) = (0~1.6):1:1. The pH of the mixture was adjusted to 7 using aqueous ammonia, followed by stirring at room temperature for 2 h. Finally, the precursor solution was heated in a muffle furnace under an air atmosphere at 200 °C with 5 °C/min to trigger the self-propagating combustion reaction, resulting in the in situ synthesis of the C-BiVO_4_ yellow pigment.

### 3.2. Characterization

#### 3.2.1. X-Ray Diffraction (XRD)

The crystal structures of the prepared pigments were analyzed using an X-ray diffractometer (Bruker D8 Advance, Karlsruhe, Germany) equipped with Cu Kα radiation (λ = 1.5406 Å). The diffraction patterns were collected in the 2θ range of 10–70° with a scanning rate of 5° min^−1^ and a step size of 0.02°. Prior to measurement, the powder samples were finely ground and uniformly spread on a low-background sample holder. The Rietveld analysis of the XRD pattern was calculated by the Fullprof-2k software (Version 2.05) package.

#### 3.2.2. X-Ray Photoelectron Spectroscopy (XPS)

The surface chemical states and elemental compositions of the samples were investigated using an X-ray photoelectron spectrometer (Thermo Fisher Scientific K-Alpha, Waltham, MA, USA) with monochromatic Al Kα radiation (hν = 1486.6 eV). The binding energies were calibrated using the C 1s peak at 284.8 eV as the reference. All spectra were deconvoluted using Gaussian-Lorentzian fitting after Shirley background subtraction.

#### 3.2.3. Electron Microscopy

The morphology and microstructure of the pigments were characterized using scanning electron microscopy (SEM, Phenom, Rotterdam, The Netherlands) and high-resolution transmission electron microscopy (HRTEM, FEI Talos F200S, Thermo Fisher Scientific, Waltham, MA, USA). For SEM analysis, the powder samples were coated with a thin conductive carbon layer prior to observation. For TEM characterization, the samples were ultrasonically dispersed in ethanol for 15 min, and a drop of the suspension was deposited onto a carbon-coated copper grid and dried naturally before measurement.

#### 3.2.4. Raman and FTIR Spectroscopy

Raman spectra were recorded using a Raman spectrometer (in Via, Renishaw, Wotton-under-Edge, UK) with a 532 nm laser excitation source in the range of 100–1200 cm^−1^. Fourier transform infrared (FTIR) spectra were collected using an FTIR spectrometer (INVENIO S, Bruker, Germany) in the range of 400–4000 cm^−1^ employing the KBr pellet method, with a sample-to-KBr mass ratio of 1 mg to 80 mg.

#### 3.2.5. BET Surface Area

N_2_ adsorption–desorption isotherms of the samples were measured using an ASAP 2020 system (Micromeritics Instrument Corporation, Norcross, GA, USA) at 77 K. Prior to measurement, the samples were degassed under vacuum to remove physically adsorbed impurities. The specific surface area (SSA) was calculated using the Brunauer-Emmett-Teller (BET) method.

#### 3.2.6. Photoluminescence and UV-Vis Diffuse Reflectance Spectroscopy

Photoluminescence (PL) spectra were obtained using a Hitachi F-7000 fluorescence spectrophotometer (Hitachi High-Tech Corporation, Tokyo, Japan) with an excitation wavelength of 325 nm. UV-vis diffuse reflectance spectra (DRS) were measured using a Lambda 850 spectrophotometer (PerkinElmer, Shelton, CT, USA) over the wavelength range of 380–800 nm. BaSO_4_ was used as the reference material. The band gap energies were estimated using the Kubelka-Munk transformation.

#### 3.2.7. Colorimetric Measurements

The colorimetric properties of the pigments were evaluated using a WSD-3C colorimeter (KANGGUANG, Beijing, China) under standard daylight illumination conditions. The measured values were expressed in the CIE-L*a*b* color space, where L* represents lightness, a* represents the red-green coordinate, and b* represents the yellow-blue coordinate. Each sample was measured at least three times at different positions, and the average values were calculated to ensure reproducibility.

### 3.3. Bacterial Inhibition Assays

The antibacterial activity was evaluated using *Escherichia coli* (*E. coli*, ATCC 8739) and *Staphylococcus aureus* (*S. aureus*, CMCC 26003). Cells were cultured overnight in 50 mL of Luria–Bertani (LB) broth at 37 °C (*E. coli*) and 25 °C (*S. aureus*) with shaking (180 rpm). The cells were harvested by centrifugation, washed twice with sterile 0.9% saline solution, and resuspended for subsequent use. The bacterial concentration was adjusted to an optical density at 600 nm (OD_600_) of approximately 0.5 using a UV–visible spectrophotometer (SDPTOP UV2900). To initiate the assay, a specific volume of bacterial suspension was transferred into a 250 mL Erlenmeyer flask containing the test materials (final concentration: 150 μg/mL). For the light-irradiated groups, the flasks were placed on a shaker approximately 20 cm below a solar simulator (equipped with a 420 nm cut-off filter), ensuring a constant light intensity. Corresponding control and non-irradiated groups were maintained in the dark. After 30 min of treatment, aliquots were collected from each flask for plate counting. The agar plates were incubated at 37 °C for 12 h, after which the colonies were quantified. The results were expressed as colony-forming units per milliliter (CFU/mL). All experiments were performed in triplicate.

## 4. Conclusions

In summary, C-BiVO_4_ yellow pigments were successfully fabricated via a one-step self-propagating combustion method using citric acid as a fuel and carbon source. Carbon doping induces lattice distortion and oxygen vacancy formation, narrows the band gap, and effectively suppresses photogenerated charge carrier recombination. At an optimal citric acid to BiVO_4_ molar ratio of 1.2, the pigment achieves outstanding chromatic performance (b* = 79.71). The C-BiVO_4_ pigment exhibits high photocatalytic degradation efficiency and excellent antibacterial activity under visible light, achieving a 99.99% inactivation rate against *E. coli*. Biocompatibility tests confirm its non-irritating nature and low acute dermal toxicity. Furthermore, the pigment demonstrates good processability in plastic and glass matrices, highlighting its potential for multifunctional coatings, packaging materials, and consumer products.

## Figures and Tables

**Table 1:** Rietveld refinement results of samples with different C-doping amounts.

n(CA)/n(Bi)	0	1.2
a (Å)	5.1814	5.1824
b (Å)	5.1033	5.1113
c (Å)	11.7019	11.7042
Volume (Å)	309.418	310.027
R_wp_ (%)	6.53	6.56
R_p_ (%)	5.00	4.96

## Data Availability

The original contributions presented in this study are included in the article/Supplementary Material. Further inquiries can be directed to the corresponding authors.

## Supplementary Materials

The following supporting information can be downloaded at https://www.mdpi.com/article/10.3390/molecules31122141/s1, Figure S1: XRD patterns of C-BiVO_4_ pigments prepared by different heating rates (a) and atmosphere (b); Figure S2: TEM (a,b) and HRTEM (c) images of the C-BiVO_4_ sample; Figure S3: Recycling experiments of photocatalytic inactivation of *S. aureus* (a) and *E. coli* (b); Table S1: CIE coordinates of C-BiVO_4_ yellow pigment prepared by different heating rates and atmosphere; Table S2: CIE coordination of C-BiVO_4_ yellow pigment prepared by different n(CA)/n(Bi); Table S3: Skin irritation scoring record of C-BiVO_4_ yellow pigment in rabbits. Table S4: Results of acute dermal toxicity test of BiVO_4_ yellow pigments in SD rats.
