# Supplementary material for: One-Step Combustion Synthesis of Carbon-Doped BiVO4 Yellow Pigments with Enhanced Visible-Light Photocatalytic Antibacterial Performance
Source: Molecules. 2026 Jun 17;31(12):2141. doi: 10.3390/molecules31122141 (PMC13306050; doi:10.3390/molecules31122141)
Supplement: Supplementary file 1 [file molecules-31-02141-s001.zip › molecules-4312445-supplementary.pdf]

Supporting Information

of

One-Step Combustion Synthesis of Carbon-Doped $BiVO_4$ Yellow Pigments with Enhanced Visible-Light Photocatalytic Antibacterial Performance

Xiaojun Zhang [1,2,*,†], Tianxu Wang [3,†], Feng Jiang [3,*], Xiaoli Su [3,*], Xun Liu [2], Yanqiao Xu [1], Guo Feng [1] and Qian Wu [1]

[1] *National Engineering Research Center for Domestic & Building Ceramics, Jingdezhen Ceramic University, Jingdezhen 333000, China; xuyanqiao@jci.edu.cn (Y.X.); fengguo@jci.edu.cn (G.F.); wuqian@jci.edu.cn (Q.W.)*

[2] *State Key Laboratory of Advanced Environmental Technology & Guangdong Provincial Key Laboratory of Mineral Physics and Materials, Guangzhou Institute of Geochemistry, Chinese Academy of Sciences, Guangzhou 510640, China; liuxun@gig.ac.cn*

[3] *Department of Material Science and Engineering, Jingdezhen Ceramic University, Jingdezhen 333000, China; 13673104706@163.com*

Correspondence: zhangxiaojun@jci.edu.cn (X.Z.); jiangfeng@jci.edu.cn (F.J.); suxiaoli@jci.edu.cn (X.S.)

[†] *These authors contributed equally to this work.*

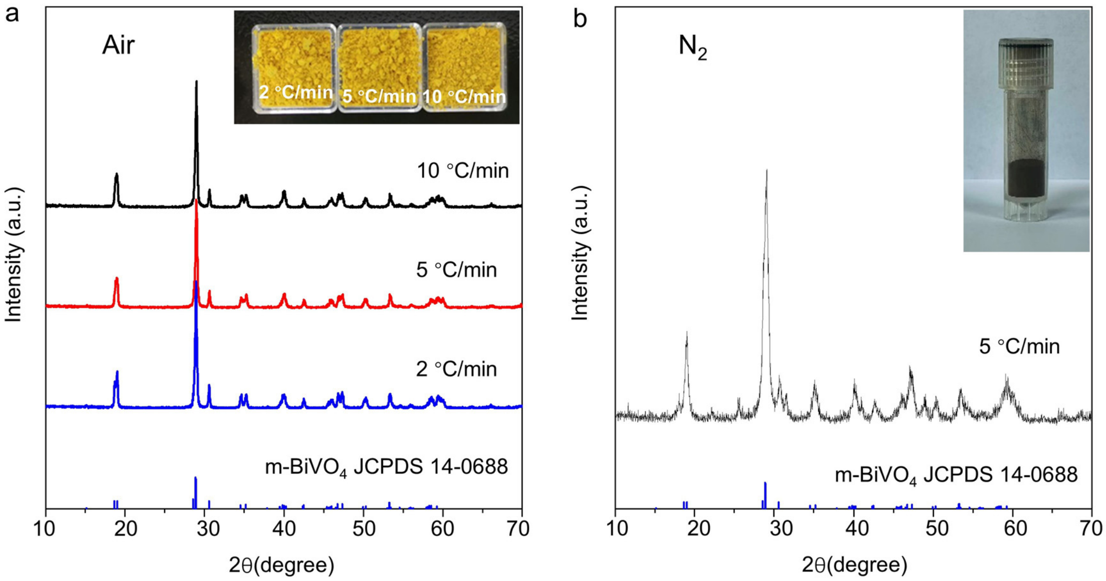

Figure S1 XRD patterns of C-$BiVO_4$ pigments prepared by different heating rates (a) and atmosphere (b)

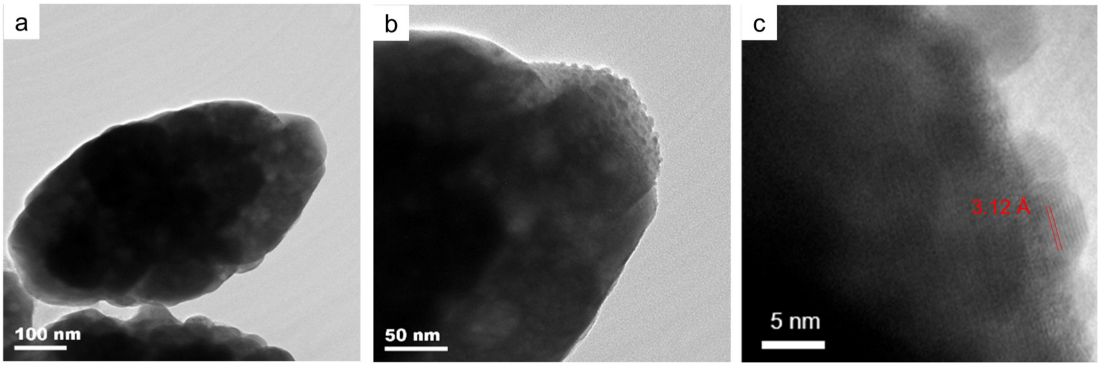

Figure S2. TEM (a,b) and HRTEM (c) images of the C-$BiVO_4$ sample.

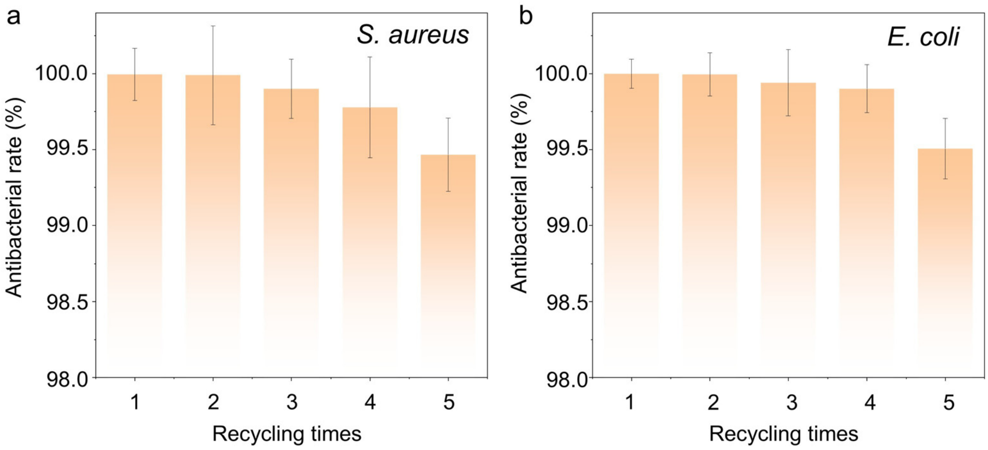

Figure S3 Recycling experiments of photocatalytic inactivation of *S. aureus* (a) and *E. coli* (b)

Table S1 CIE coordinates of C-$BiVO_4$ yellow pigment prepared by different heating rates and atmosphere

Atmosphere	Heating rate	CIE		
		*L**	*a**	*b**
air	2	76.63	6.34	77.21
air	5	74.07	7.78	79.71
air	10	73.40	7.98	72.07
nitrogen	5	23.39	9.81	12.98

Table S2 CIE coordination of C-$BiVO_4$ yellow pigment prepared by different n(CA)/n(Bi)

Citric acid/$BiVO_4$	CIE		
	*L**	*a**	*b**
0	68.81	12.76	68.20
0.2	73.34	8.09	68.36
0.4	76.22	6.39	72.48
0.6	77.20	3.96	74.33
0.8	73.02	8.64	75.03
1.0	75.24	4.17	77.61
1.2	74.07	7.78	79.71
1.4	74.51	6.42	78.87
1.6	76.73	2.67	74.20

Table S3 Skin irritation scoring record of C-$BiVO_4$ yellow pigment in rabbits

Group	24 h (Test/Control)	48 h (Test/Control)	72 h (Test/Control)
	Erythema/ Edema	Erythema/ Edema	Erythema/ Edema
1	0, 0 / 0, 0	0, 0 / 0, 0	0, 0 / 0, 0
2	0, 0 / 0, 0	0, 0 / 0, 0	0, 0 / 0, 0
3	0, 0 / 0, 0	0, 0 / 0, 0	0, 0 / 0, 0
4	0, 0 / 0, 0	0, 0 / 0, 0	0, 0 / 0, 0
Mean score	0 / 0	0 / 0	0 / 0

Table S4 Results of acute dermal toxicity test of $BiVO_4$ yellow pigments in SD rats

Sex	Dosage (mg/kg)	Number of animals	Number of deaths	LD_{50}（mg/kg）
Male	2000	10	0	>2000
Female	2000	10	0	>2000
